# DSM-5 non-suicidal self-injury disorder in a community sample: comparing NSSI engagement, recency and severity among emerging adults

**DOI:** 10.3389/fpsyt.2023.1251514

**Published:** 2023-12-08

**Authors:** Serafine Dierickx, Laurence Claes, Tinne Buelens, Dirk Smits, Glenn Kiekens

**Affiliations:** ^1^Faculty of Psychology and Educational Sciences, KU Leuven, Leuven, Belgium; ^2^Expertise Unit Resilient People, University Colleges Leuven-Limburg, Leuven, Belgium; ^3^Faculty of Medicine and Health Sciences (CAPRI), University of Antwerp, Antwerp, Belgium; ^4^Department of Clinical Psychology, University of Amsterdam, Amsterdam, Netherlands; ^5^Research Department, Odisee University of Applied Sciences, Brussel, Belgium; ^6^Department of Neurosciences, Center for Contextual Psychiatry, KU Leuven, Leuven, Belgium; ^7^Department of Medical and Clinical Psychology, Tilburg University, Tilburg, Netherlands

**Keywords:** non-suicidal self-injury, recency, severity, non-suicidal self-injury disorder, emerging adults

## Abstract

Up to one in five emerging adults engage in non-suicidal self-injury (NSSI). Providing a better understanding of factors that differentiate between who engages in lifetime NSSI and who is more likely to engage in recent and clinically severe NSSI can provide meaningful information for prevention and intervention of NSSI. The present study (*n* = 669) considered NSSI lifetime engagement (no prior history of NSSI vs. lifetime NSSI), recency [past NSSI (>12 months ago) vs. recent (≤12-month) NSSI], and clinical severity among those with recent NSSI (subthreshold vs. DSM-5 NSSI disorder). The prevalence of NSSI disorder was 8.4% in emerging adults aged 18 to 26 years old. Higher anxiety levels were related to NSSI engagement, but only depressive symptoms and NSSI versatility were consistently associated with more recent NSSI and NSSI disorder. A stepped-care approach may be required in addressing NSSI among emerging adults.

## 1 Introduction

Non-Suicidal Self-Injury (NSSI), which refers to direct and deliberate damage to an individual’s own bodily tissue without suicidal intent (1), is a major mental health challenge among emerging adults (2, 3). In accordance with the Diagnostic and Statistical Manual of Mental Disorders (DSM-5-TR) (4), we use the term NSSI, which excludes self-injury with suicidal intent. If there is some intent to die present, it is classified as a suicide attempt. In contrast, the term ‘self-harm,’ still often used in British literature (5), encompasses both non-suicidal and suicidal self-injurious behaviors. Emerging adulthood (i.e., 18–25 years old) is typically described as the developmental period between adolescence and young adulthood which is marked by increased exploration and psychosocial risk-taking, but also vulnerability towards NSSI (6, 7). Among first-year college students (age 18–20; *n* = 20,842), the lifetime and 12-month prevalence of NSSI is estimated at one in five and one in 10, respectively (2). Age of NSSI onset is most often situated in mid-adolescence (age 14 to 16), with a second peak during emerging adulthood (8). More than half of the adolescents with a history of NSSI persist to self-injure during emerging adulthood (8, 9), but the probability of desisting NSSI is also highest at ages 18 to 21 (10). Emerging adults reporting recent and clinically considered severe NSSI are at higher risk for adverse outcomes such as mental disorders, suicidal thoughts and behaviors, and suicide attempts than peers who engaged in NSSI in the past (9, 11), which underscores the importance of effective differentiation among those with a lifetime history of NSSI. Considering severity, the NSSI Disorder (NSSI-D) was added to the fifth edition of the Diagnostic and Statistical Manual of Mental Disorders (DSM-5) (12). To meet a diagnosis for NSSI-D, individuals need to report NSSI engagement at least 5 days during the past year, with NSSI causing significant distress or severe impairment across life domains (12). Other diagnostic criteria include affective or social precipitants, NSSI urges, and the expectation that NSSI will result in relief, resolve interpersonal difficulties, or increase positive feelings. Prevalence of NSSI-D is estimated at 0.2–0.8% among emerging adults, with higher rates in women than men (13, 14). In line with prior studies (2, 9), providing a better insight into factors that differentiate individuals who engage in lifetime NSSI (vs. no NSSI) and who are more likely to engage in recent (≤ 12 months vs. > 12 months ago) and clinically severe NSSI among those with recent NSSI (subthreshold vs. NSSI-D) can provide meaningful information for prevention and intervention strategies that target NSSI. Namely, (a) differentiation in the presence/absence of lifetime NSSI engagement can tell us more about risk factors for the onset of NSSI, which can feed general primary care and preventative initiatives; (b) differentiation between recency of NSSI (≤ 12 months vs. > 12 months ago) is important for secondary care and therapeutic interventions; and (c) differentiation between severity (subthreshold NSSI vs. NSSI-D) can improve insights of tertiary or specialized care.

Overall, studies have shown higher levels of depression, anxiety, disruptive behaviors, personality dysfunctioning and anger, and lower levels of self-esteem (13, 15) in individuals who engage in NSSI compared to those who do not (i.e., lifetime NSSI engagement vs. no NSSI). Prior work also suggests that higher internalized anger expression (compared to externalized anger expression) is associated with an increased risk of NSSI (16). However, few studies have examined whether these clinical constructs might also help to differentiate between those who are more likely to engage in recent (≤ 12 months vs. > 12 months ago) and clinically severe NSSI among those with recent NSSI (subthreshold vs. NSSI-D) (2, 15). There is some evidence that individuals who engage in 12-month NSSI report higher levels of negative affect, rumination, self-criticism, personality dysfunctioning, severe life stress and impairment, and mood disorders compared to those with past NSSI or compared to those who never engaged in NSSI (9, 13, 17–19). NSSI recency has also been positively associated with an earlier onset (11 years or younger, compared to 14–17 years old) and with onset in the year before college (2). Finally, a handful of studies investigated DSM-5 NSSI-D and observed that individuals meeting NSSI-D criteria reported increased NSSI versatility (i.e., the number of different NSSI methods), higher levels of psychopathology and significantly more impaired functioning than individuals who self-injured in the past year but do not meet the NSSI-D criteria (i.e., subthreshold NSSI) (20). Yet, given that up to one in five emerging adults engage in NSSI, more work is required considering meaningful differences among those with a lifetime history of NSSI (2, 5, 21). Obtaining a clearer picture regarding the unique clinical correlates and potential risk factors of NSSI engagement, recency, and severity would provide valuable information for prevention efforts and clinical assessment.

To help address this gap in the literature, the present study compares emerging adults with and without a lifetime NSSI history (i.e., engagement), recent vs. past NSSI (recency: ≤ 12 months vs. > 12 months ago), and subthreshold NSSI vs. NSSI-D with respect to gender, age of NSSI onset, NSSI versatility, anxiety, depression, personality dysfunctioning, self-esteem, anger-in and anger-out (i.e., internalized and externalized anger expression, respectively). We expect individuals with recent NSSI and NSSI-D to report more NSSI versatility, either early or pre-college onset (i.e., 13 years or younger; or 17 years or older), more anxious and depressive symptoms, more personality dysfunctioning, more internalized (i.e., self-critical) anger expression, and less self-esteem than individuals with past NSSI and subthreshold NSSI, respectively. Given that theory (e.g., the Benefits and Barriers model (21) suggests that negative mood is an “affective engine” that drives repeated NSSI) and empirical literature suggests that negative mood uniquely increases risk for persistent NSSI (9, 13, 14, 17, 18), we expect that individuals with more severe depressive symptomatology will be more likely to report NSSI engagement, recent NSSI, and NSSI-D. In addition, we anticipate meaningful differences between individuals with past and subthreshold NSSI and those with NSSI-D.

## 2 Methods

Data were collected through an anonymous web-based survey using convenience sampling. The sample comprised 669 emerging adults (*M*_age_ = 21.48; SD = 2.20; range 18 to 26 years), of whom 205 participants (30.64%) identified as men and 454 (69.36%) as women. The study was approved by SMEC KU Leuven. Invitations to participate in an anonymous web-based survey were sent to socio-cultural organizations (e.g., sports clubs and music societies) to distribute among their Dutch-speaking emerging adult members. There was no reimbursement for participation in the study. The study took about 20 min.

The data collection comprised socio-demographic variables (i.e., age and gender) and self-report questionnaires. Participants who reported lifetime NSSI (dichotomous item) completed a follow-up questionnaire (22) evaluating NSSI methods, age of onset, and NSSI-D criteria (KR-20 = 0.73). Consistent with prior work that considered heterogeneity in engagement, recency and severity of NSSI (14, 22), we differentiated between emerging adults with no history of NSSI vs. lifetime NSSI (engagement); recent vs. past NSSI (recency: ≤ 12 months vs. > 12 months ago) and severity of NSSI among those with recent NSSI (subthreshold NSSI vs. NSSI-D).

Anxiety and depression were evaluated using the Anxiety (10 items; α = 0.91) and Depression (16 items; α = 0.94) subscales of the Symptom Checklist-Revised (SCL-90-R) (23). The items were scored on a 5-point Likert scale ranging from 1 (Not at all) to 5 (Extremely). Higher anxiety or depression scores reflected more severe anxiety and depressive symptoms, respectively. Personality dysfunctioning was assessed using the Dutch Five-Item Screening Scale for Personality Disorders (FISSPD; α = 0.83) (24). Participants were asked to report to what extent they agree with five items on a 5-point Likert scale ranging from 0 (Completely disagree) to 4 (Completely agree). The mean score of the five items resulted in a single severity score with a higher score indicating increasing severity of personality dysfunctioning. Self-esteem (10 items; α = 0.89) was measured using the Rosenberg Self-Esteem Scale (25). Participants were asked to rate their agreement with 10 items on a 4-point Likert scale ranging from 0 (Strongly agree) to 3 (Strongly disagree). Higher scores on the total scale indicated more self-esteem. Lastly, internalized and externalized anger expression were assessed using the anger-in (8 items; α = 0.71) and anger-out (8 items; α = 0.78) subscales of the State Trait Anger Expression Inventory 2 (STAXI-2) (26). Participants self-reported the frequency with which anger is expressed on a 4-point Likert scale ranging from 1 (Almost never) to 4 (Almost always). Higher scores on anger-in or anger-out reflect more internalized or externalized anger expression, respectively.

A series of binary logistic regression models was estimated to understand the association between the clinical constructs and NSSI engagement (no NSSI vs. lifetime NSSI), NSSI recency (recency: ≤ 12 months vs. > 12 months ago), and NSSI severity (subthreshold vs. NSSI-D). For each comparison, we conducted bivariate (controlling for gender) and multivariate models (including all covariates simultaneously) with two-sided significance tests. Correlates that remain significant throughout the multivariate analyses will be compared using norm scores, if available.

## 3 Results

**Figure 1:**
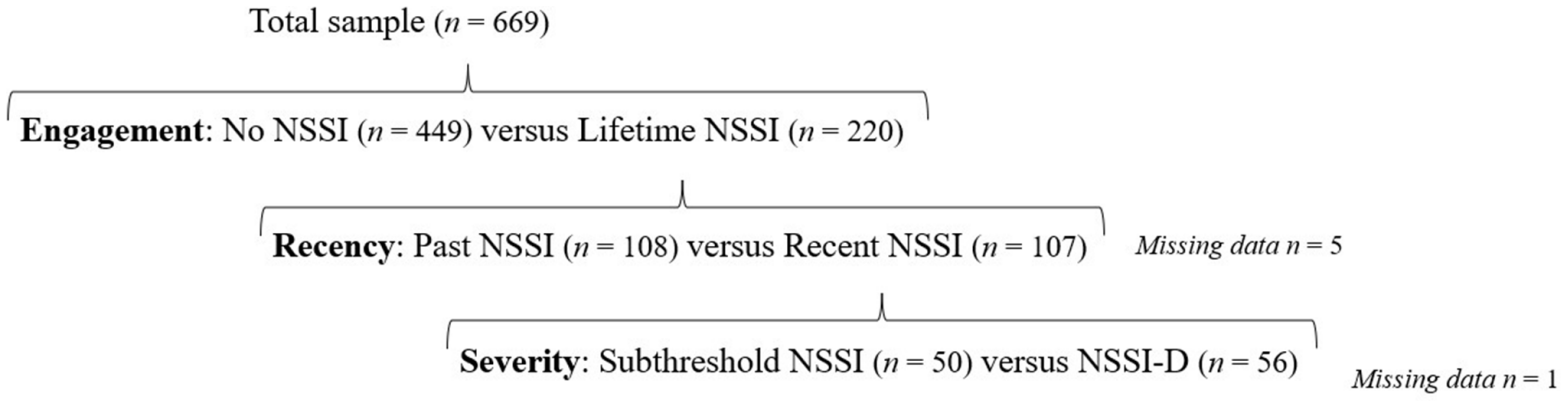
Data differentiation in engagement, recency and severity of NSSI.

Lifetime and 12-month prevalence of NSSI was 32.9 and 16.0%, respectively. Prevalence of DSM-5 NSSI-D was 8.4%. There were 449 emerging adults with no prior history of NSSI and 220 individuals with a lifetime history of NSSI. Of these, 108 reported past NSSI (>12 months) and 107 recent NSSI (≤ 12 months). Of those with recent NSSI, 50 individuals reported subthreshold NSSI and 56 met criteria for NSSI-D. Five participants (0.75%) could not be classified due to missing data. The groups are visualized in Figure 1.

When controlling for gender, higher anxiety and depression, increased personality dysfunctioning and anger-in, and decreased levels of self-esteem increased odds for lifetime NSSI engagement (compared to no NSSI). As can be seen in Table 1, higher anxiety and depression remained independently associated with NSSI engagement in a multivariate model controlling for all clinical correlates. Similarly, higher anxiety and depression, personality dysfunctioning and anger-in, and decreased self-esteem were significantly associated with increased odds of recent NSSI (≤ 12 months) compared to those with past NSSI. Individuals with recent NSSI also reported greater NSSI versatility than those who self-injured more than 12 months ago. Multivariate models showed that NSSI versatility and higher depression levels were independently associated with NSSI recency when controlling for all clinical correlates. In addition, reporting an age of onset of NSSI after 17 years of age became a significant correlate of recent NSSI in this multivariate model. Finally, we evaluated associations among those with NSSI-D (compared to subthreshold NSSI), and found that greater NSSI versatility and higher anxiety and depression, personality dysfunctioning, and anger-in were associated with increased odds of NSSI-D; whereas self-esteem was associated with decreased odds of NSSI-D. However, only NSSI versatility and depressive symptoms remained independently associated with NSSI-D in the multivariate model.

**Table 1:** Bivariate and multivariate logistic regression analyses examining NSSI behavior.

		Engagement: No NSSI versus lifetime NSSI history across the sample (*n* = 669)^a^		Recency: Past (i.e., > 12-months) versus recent (≤ 12-months) NSSI among those with lifetime NSSI history (*n* = 215)		Severity: Subthreshold (i.e., 12-month NSSI without disorder) versus DSM-5 NSSI disorder among those with recent NSSI (*n* = 106)	
		Bivariate analyses	Multivariate analyses	Bivariate analyses	Multivariate analyses	Bivariate analyses	Multivariate analyses
		OR (95% CI)	OR (95% CI)	OR (95% CI)	OR (95% CI)	OR (95% CI)	OR (95% CI)
Being woman		**1.61 (1.12–2.32)** ^*^	1.28 (0.85–1.92)	1.21 (0.65–2.26)	0.72 (0.31–1.68)	1.16 (0.47–2.88)	0.75 (0.21–2.69)
Age of NSSI onset^b^							
	13 or younger			*(ref)*	*(ref)*	*(ref)*	*(ref)*
	13 to 17			1.05 (0.51–2.16)	1.39 (0.54–3.57)	1.38 (0.47–4.08)	1.86 (0.49–7.03)
	17 or older			1.76 (0.66–4.70)	**4.05 (1.15–14.28)** ^*^	0.91 (0.23–3.67)	5.05 (0.78–32.79)
NSSI versatility				**1.84 (1.50–2.26)** ^***^	**1.92 (1.49–2.46)** ^***^	**1.50 (1.18–1.91)** ^***^	**1.77 (1.26–2.49)** ^***^
SCL-90-R							
	Anxiety	**1.08 (1.06–1.11)** ^***^	**1.03 (1.00–1.07)** ^*^	**1.07 (1.03–1.10)** ^***^	0.97 (0.92–1.03)	**1.07 (1.02–1.12)** ^**^	1.01 (0.94–1.10)
	Depression	**1.05 (1.04–1.06)** ^***^	**1.02 (1.00–1.05)** ^*^	**1.07 (1.04–1.09)** ^***^	**1.06 (1.02–1.11)** ^**^	**1.07 (1.03–1.10)** ^***^	**1.08 (1.01–1.15)** ^*^
FISSPD							
	Personality dysfunctioning	**1.92 (1.59–2.30)** ^***^	1.13 (0.85–1.48)	**2.04 (1.48–2.81)** ^***^	0.98 (0.59–1.65)	**1.75 (1.12–2.72)** ^*^	0.76 (0.36–1.61)
RSE							
	Self-esteem	**0.31 (0.22–0.43)** ^***^	0.66 (0.42–1.02)	**0.27 (0.15–0.49)** ^***^	0.73 (0.32–1.69)	**0.35 (0.15–0.78)** ^*^	1.14 (0.34–3.85)
STAXI-2							
	Anger-in	**1.06 (1.02–1.10)** ^**^	0.98 (0.93–1.02)	**1.07 (1.00–1.14)** ^*^	1.00 (0.91–1.10)	**1.14 (1.04–1.25)** ^**^	1.11 (0.97–1.27)
	Anger-out	1.04 (1.00–1.08)	1.01 (0.97–1.06)	1.06 (0.99–1.13)	1.04 (0.96–1.14)	1.03 (0.95–1.12)	1.01 (0.90–0.14)
Nagelkerke R^2^			0.19		0.45		0.41

^*^p < 0.05, ^**^p < 0.01, ^***^p < 0.001. Significant odds ratios are made boldface.

a For No NSSI versus lifetime NSSI, Age of NSSI onset and NSSI versatility were excluded.

b To avoid overlap with recent NSSI, individuals who reported age of onset ≤ 12 months ago were excluded from the bivariate model involving Age of NSSI onset.

OR, Odds Ratio; NSSI, Non-Suicidal Self-Injury; SCL-90-R, Revised Symptom Checklist; FISSPD, Five-Item Screening Scale for Personality Disorders; RSE, Rosenberg Self-Esteem; STAXI-2, State–Trait Anger Expression Inventory 2.

To evaluate meaningful differences in depression scores between groups, we used the norm scores of the Dutch community population. This revealed that individuals without NSSI engagement (17.23; SD = 13.60) scored low-to-below-average for depression (SCL-90-R), those that reported past NSSI scored average-to-above-average (22.07; SD = 14.01), whereas individuals with subthreshold (29.92; SD = 14.09), and NSSI-D (40.25; SD = 11.74) scored high and very high, respectively. A one-way post-hoc ANOVA confirmed that the differences for depression between these groups were significant [*F*(3, 659) = 56.58, *p* < 0.001], with significant incremental increases across all groups (Tukey post-hoc). Considering NSSI versatility, there were no norm scores available. Mean number of different NSSI methods for the past NSSI, subthreshold NSSI, and NSSI-D groups were 2.1 (SD = 1.3), 3.0 (SD = 1.6), and 4.3 (SD = 1.9), respectively. The differences in average NSSI versatility between groups were again significant [*F*(2, 211) = 36.82, *p* < 0.001] with significant incremental increases for the past NSSI, the subthreshold NSSI and the NSSI-D group (Tukey post-hoc).

## 4 Discussion

The present study examined differentiated meaningful epidemiological NSSI outcomes (i.e., engagement, recency, and severity) and investigated associations with respect to clinical symptomatology: being (a) no NSSI vs. lifetime NSSI (engagement), (b) past vs. 12-month NSSI (recency) among those with lifetime NSSI, and (c) subthreshold NSSI vs. NSSI-D among those with recent NSSI (severity). Findings suggest that both anxiety and depression are associated with NSSI engagement, but only depression was consistently associated with more recent and clinically severe NSSI. Personality dysfunctioning, self-esteem and anger-in also showed associations across the comparison groups but became nonsignificant when depression was taken into account. This pattern of findings aligns with Hooley and Franklin (21) Benefits and Barriers Model, which defines emotional distress as the maintaining factor for NSSI after its onset.

We found a rate of 8.4% for NSSI-D in the present sample, which is considerably higher than in prior work (i.e., 0.2–0.8%; 12,26). One potential reason for this might be because the study was conducted during the COVID-19 pandemic (27). In line with Ammerman et al. (28), higher NSSI versatility was associated with NSSI recency and severity. Due to habituation, individuals may need to engage in different methods of NSSI (i.e., increased NSSI versatility) to experience the same emotion-regulatory effect (29). Importantly, individuals using different methods of NSSI are also more likely to report suicidal thoughts and behaviors (30).

Notably, we identified depression as a consistent, non-specific correlate of recent and severe NSSI among emerging adults, with the highest symptoms found among individuals meeting the criteria for NSSI-D. The present study cannot indicate the direction of this relationship, with prior work indicating that this relationship might be bi-directional (2). In addition, NSSI and depression may also have shared risk factors (e.g., trauma, emotion regulation difficulties) (31), suggesting that a unified approach for general primary care and preventative initiatives (32), in which interventions aiming at shared risk factors, may be useful to prevent both the onset of NSSI and depression.

Our findings support a stepped-care approach in which NSSI is addressed both dimensionally and categorically across different levels of care (33). The dimensional level comprises treating NSSI as a behavior that occurs on a continuum that considers engagement, recency, and severity when matching interventions to individuals’ needs. Considering engagement, it seems important for prevention and general care to target general risk factors such as anxiety and depression. For individuals who have engaged in past NSSI and those engaging in recent NSSI, it could be advised to discuss NSSI in therapy and explore the factors that facilitate desistance of the behavior. Emerging adults who have engaged in NSSI in the past should thus not be left without support, as the study results also point out that these individuals report significantly more anxious and depressive symptoms compared to individuals who have never engaged in NSSI. This is in line with recent work that NSSI recovery constitutes more than just behavioral cessation and involves also discussion around ongoing thoughts and ambivalence about stopping NSSI (34–36). Finally, the categorical approach includes identifying individuals who meet the NSSI-D criteria and might require specialized treatment that involve a targeted focus on NSSI (e.g., dialectical behavior therapy). The merits of a stepped-care approach were recently also acknowledged by DSM-5’s recent text revision (4) which mentions NSSI as a behavior that may warrant ongoing clinical attention in its own right.

The current study’s results should be interpreted considering the following limitations. The study design was cross-sectional in nature. This implies that the findings should be replicated in future prospective research to consider the directionality between constructs. Additionally, the data collection was based on convenience sampling, which may lead to sampling bias and implies that prevalence rates should be interpreted with caution. Further, the study consisted of only self-report questionnaires, which can result in reporting bias and shared method variance. Even though the NSSI-D criteria (4) and other clinical constructs that were included were assessed using well-validated instruments, future work should consider assessing NSSI disorder criteria using a golden standard diagnostic interview (3, 20). These limitations notwithstanding, this study shows that depressive symptoms and NSSI versatility are consistently associated with more recent and more severe NSSI among emerging adults who self-injure. These findings highlight the need to capture meaningful differences among emerging adults reporting engagement in self-injury by considering the recency and severity of NSSI routinely in future research studies.

## Data availability statement

The raw data supporting the conclusions of this article will be made available by the authors, without undue reservation.

## Ethics statement

The studies involving humans were approved by Social and Societal Ethics Committee (Katholieke Universiteit Leuven, Belgium) under file number G-2021-3870-R2 (MAR). The studies were conducted in accordance with the local legislation and institutional requirements. The participants provided their written informed consent to participate in this study.

## Author contributions

SD, LC, TB, DS, and GK contributed to conceptualization and design of the study. SD organized the database and wrote the original draft of the manuscript. SD and GK performed the statistical analysis. All authors contributed to the article and approved the submitted version.

## References

[1] International Society for the Study of self-injury. What is self-injury? (2018). Available at: https://itriples.org/about-self-injury/what-is-self-injury/.

[2] Kiekens G Hasking P Bruffaerts R Alonso J Auerbach RP Bantjes J. Non-suicidal self-injury among first-year college students and its association with mental disorders: results from the world mental health international college student (WMH-ICS) initiative. Psychol Med. (2021) 53:875–86. doi: 10.1017/s0033291721002245 34140062 PMC8683565

[3] Swannell SV Martin GE Page A Hasking P St John NJ. Prevalence of nonsuicidal self-injury in nonclinical samples: systematic review, meta-analysis and meta-regression. Suicide Life Threat Behav. (2014) 44:273–303. doi: 10.1111/sltb.12070 24422986

[4] American Psychiatric Association. Diagnostic and statistical manual of mental disorders, text revision (DSM-5-TR). 5th ed (2022) text revision.

[5] Hooley JM Fox KR Boccagno C. Nonsuicidal self-injury: diagnostic challenges and current perspectives. Neuropsychiatr Dis Treat. (2020) 16:101–12. doi: 10.2147/ndt.s198806 32021203 PMC6959491

[6] Nelson LJ. The theory of emerging adulthood 20 years later: a look at where it has taken us, what we know now, and where we need to go. Emerg Adulthood. (2021) 9:179–88. doi: 10.1177/2167696820950884

[7] Kiekens G Hasking P Claes L Boyes M Mortier P Auerbach RP. Predicting the incidence of non-suicidal self-injury in college students. Eur Psychiatry. (2019) 59:44–51. doi: 10.1016/j.eurpsy.2019.04.002 31035219

[8] Gandhi A Luyckx K Baetens I Kiekens G Sleuwaegen E Berens A. Age of onset of non-suicidal self-injury in Dutch-speaking adolescents and emerging adults: an event history analysis of pooled data. Compr Psychiatry. (2018) 80:170–8. doi: 10.1016/j.comppsych.2017.10.007 29121554

[9] Kiekens G Claes L Hasking P Mortier P Bootsma E Boyes M. A longitudinal investigation of non-suicidal self-injury persistence patterns, risk factors, and clinical outcomes during the college period. Psychol Med. (2022) 53:6011–26. doi: 10.1017/s0033291722003178 36325723

[10] Turner BJ Helps CE Ames ME. Stop self-injuring, then what? Psychosocial risk associated with initiation and cessation of nonsuicidal selfinjury from adolescence to early adulthood. J Psychopathol Clin Sci. (2022) 131:45–57. doi: 10.1037/abn0000718 34843270

[11] Hamza CA Willoughby T. Nonsuicidal self-injury and suicidal risk among emerging adults. J Adolesc Health. (2016) 59:411–5. doi: 10.1016/j.jadohealth.2016.05.019 27485906

[12] American Psychiatric Association. Diagnostic and statistical manual of mental disorders. 5th ed (2013).

[13] Benjet C González-Herrera I Castro-Silva E Méndez E Borges G Casanova L. Non-suicidal self-injury in Mexican young adults: prevalence, associations with suicidal behavior and psychiatric disorders, and DSM-5 proposed diagnostic criteria. J Affect Disord. (2017) 215:1–8. doi: 10.1016/j.jad.2017.03.025 28288307

[14] Kiekens G Hasking P Claes L Mortier P Auerbach RP Boyes M. The DSM-5 nonsuicidal self-injury disorder among incoming college students: prevalence and associations with 12-month mental disorders and suicidal thoughts and behaviors. Depress Anxiety. (2018) 35:629–37. doi: 10.1002/da.22754 29697881

[15] Cipriano A Cella S Cotrufo P. Nonsuicidal self-injury: a systematic review. Front Psychol. (2017) 8:8. doi: 10.3389/fpsyg.2017.01946 29167651 PMC5682335

[16] Cipriano A Cella S Cotrufo P. Non-suicidal self-injury among Italian adolescents: the role of parental rejection, self-concept, anger expression, and body investment. Clin Neuropsychiatry. (2020) 17:330. doi: 10.36131/cnfioritieditore20200602 34909011 PMC8629071

[17] Kiekens G Hasking P Nock M Kleiman E Kirtley OJ Houben M. A comparison of affective-cognitive dynamics in daily life between emerging adults with and without past-year non-suicidal self-injury. Behav Ther. (2022). doi: 10.31234/osf.io/a2nh3 38670662

[18] Burke TA McArthur BA Daryanani I Abramson LY Alloy LB. Latent classes of trait affect and cognitive affective regulation strategies are associated with depression, non-suicidal self-injury, and well-being. J Affect Disord. (2018) 225:180–7. doi: 10.1016/j.jad.2017.08.015 28837951 PMC5663635

[19] Eggermont K Raymaekers K Claes L Buelens T Bogaerts A Luyckx K. Impairment in personality functioning throughout adolescence and co-development with personality traits, emotion regulation strategies, and psychopathology. J Res Pers. (2023) 104:104380. doi: 10.1016/j.jrp.2023.104380

[20] Zetterqvist M. The DSM-5 diagnosis of nonsuicidal self-injury disorder: a review of the empirical literature. Child Adolesc Psychiatry Ment Health. (2015) 9:31. doi: 10.1186/s13034-015-0062-7 26417387 PMC4584484

[21] Hooley JM Franklin JC. Why do people hurt themselves? A new conceptual model of nonsuicidal self-injury. Clin Psychol Sci. (2017) 6:428–51. doi: 10.1177/2167702617745641

[22] Buelens T Luyckx K Kiekens G Gandhi A Muehlenkamp JJ Claes L. Investigating the DSM-5 criteria for non-suicidal self-injury disorder in a community sample of adolescents. J Affect Disord. (2020) 260:314–22. doi: 10.1016/j.jad.2019.09.009 31521868

[23] Arrindell WA Ettema JHM. SCL-90: Handleiding bij een multidimensionele psychopathologie-indicator [SCL-90: Manual to a multidimensional psychopathology indicator]. 2nd ed. Amsterdam: Pearson (2003).

[24] Eggermont K Luyckx K Smits D Bogaerts A Buelens T Bastiaens T. The validation of a five-item screening scale for personality disorders in Dutch-speaking community adolescents and adults. J Psychopathol Behav Assess. (2022) 44:418–31. doi: 10.1007/s10862-022-09951-1

[25] Franck E De Raedt R Barbez C Rosseel Y. Psychometric properties of the Dutch Rosenberg self-esteem scale. Psychol Belg. (2008) 48:25–31. doi: 10.5334/pb-48-1-25

[26] Lievaart M Franken IHA Hovens JE. Anger assessment in clinical and nonclinical populations: further validation of the state-trait anger expression inventory-2. J Clin Psychol. (2016) 72:263–78. doi: 10.1002/jclp.22253 26766132

[27] Zetterqvist M Landberg JLS Svedin CG. The psychosocial consequences of covid-19 in adolescents with nonsuicidal self-injury. Child Adolesc Psychiatry Ment Health. (2023) 17:33. doi: 10.1186/s13034-023-00566-2 36871031 PMC9985473

[28] Ammerman BA Jacobucci R Turner BJ Dixon-Gordon KL McCloskey MS. Quantifying the importance of lifetime frequency versus number of methods in conceptualizing nonsuicidal self-injury severity. Psychol Violence. (2020) 10:442–51. doi: 10.1037/vio0000263

[29] Tanner AK Hasking P Martin G. Suicidality among adolescents engaging in nonsuicidal self-injury (NSSI) and firesetting: the role of psychosocial characteristics and reasons for living. Child Adolesc Psychiatry Ment Health. (2015) 9:33. doi: 10.1186/s13034-015-0068-1 26421057 PMC4585995

[30] Turner BJ Layden BK Butler SM Chapman AL. How often, or how many ways: clarifying the relationship between non-suicidal self-injury and suicidality. Arch Suicide Res. (2013) 17:397–415. doi: 10.1080/13811118.2013.802660 24224673

[31] Fox KR Franklin JC Ribeiro JD Kleiman EM Bentley KH Nock MK. Meta-analysis of risk factors for nonsuicidal self-injury. Clin Psychol Rev. (2015) 42:156–67. doi: 10.1016/j.cpr.2015.09.002 26416295 PMC4772426

[32] Bentley KH. Applying the unified protocol transdiagnostic treatment to nonsuicidal self-injury and co-occurring emotional disorders: a case illustration. J Clin Psychol. (2017) 73:547–58. doi: 10.1002/jclp.22452 28221666

[33] Plener PL. Tailoring treatments for adolescents with nonsuicidal self-injury. Eur Child Adolesc Psychiatry. (2020) 29:893–5. doi: 10.1007/s00787-020-01523-6 32236748

[34] Gray N Hasking P Boyes ME. The impact of ambivalence on recovery from non-suicidal self-injury: considerations for health professionals. J Public Ment Health. (2021) 20:251–8. doi: 10.1108/JPMH-07-2020-0093

[35] Kelada L Hasking P Melvin G Whitlock J Baetens I. “I do want to stop, at least I think I do”: an international comparison of recovery from nonsuicidal self-injury among young people. J Adolesc Res. (2016) 33:416–41. doi: 10.1177/0743558416684954

[36] Lewis SP Hasking PA. Self-injury recovery: a person-centered framework. J Clin Psychol. (2020) 77:884–95. doi: 10.1002/jclp.23094 33296508

